# LncRNA TMPO‐AS1 promotes hepatocellular carcinoma cell proliferation, migration and invasion through sponging miR‐329‐3p to stimulate FOXK1‐mediated AKT/mTOR signaling pathway

**DOI:** 10.1002/cam4.3046

**Published:** 2020-05-27

**Authors:** Xiaobo Guo, Yun Wang

**Affiliations:** ^1^ Department of Hematology Xi'an Central Hospital Affiliated to Medical College of Xi'an Jiaotong University Xi'an Shaanxi China; ^2^ Department of Gastroenterology the First Affiliated Hospital of Xi'an Jiaotong University Xi'an Shaanxi China

**Keywords:** FOXK1, HCC, miR‐329‐3p, TMPO‐AS1

## Abstract

**Purpose:**

Hepatocellular carcinoma (HCC) is one of the leading causes of cancer‐related death worldwide. Numerous analyses have revealed the abnormal expression of long non‐coding RNAs (lncRNAs) in HCC cells. This study aims to explore biological functions of lncRNA TMPO‐AS1 (TMPO antisense RNA 1) in HCC cell proliferation, apoptosis, invasion and migration.

**Methods:**

The gene expression in HCC tissues and cell lines were measured by qRT‐PCR. The role of TMPO‐AS1 in HCC was confirmed by CCK‐8, colony formation, TUNEL, transwell and western blot as well as by in vivo experiments. RNA pull down and luciferase reporter assays were utilized to prove the binding relationship between TMPO‐AS1/FOXK1 (forkhead box K1) andmiR‐329‐3p. Rescue assays elucidated the regulatory effects of TMPO‐AS1/miR‐329‐3p/FOXK1/AKT/mTOR pathway on cellular activities in HCC.

**Results:**

TMPO‐AS1was upregulated in HCC tissues and cells and its depletion inhibits HCC cell proliferation, invasion, migration, and EMT process as well as tumor growth. Furthermore, TMPO‐AS1 could bind with miR‐329‐3p, which suppressed HCC cell proliferation. FOXK1 served as the target gene of miR‐329‐3p and TMPO‐AS1 upregulated FOXK1 by sponging miR‐329‐3p in HCC cells. Additionally, FOXK1 overexpression or miR‐329‐3p inhibitor neutralized the repressing effects of TMPO‐AS1 knockdown on HCC development. Finally, it verified that TMPO‐AS1 could regulate AKT/mTOR pathway via FOXK1 to promote HCC.

**Conclusion:**

TMPO‐AS1 contributes to HCC progression by sponging miR‐329‐3p to activate FOXK1‐mediated AKT/mTOR signaling pathway.

## Graphical abstract

TMPO‐AS1 was high expressed in HCC tissues and cell lines it regulates HCC cell proliferation, apoptosis and invasion. TMPO‐AS1is negatively correlated with miR‐329‐3p expression. Oncogenic function of FOXK1 in HCC cells is dependent on miR‐329‐3p.

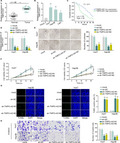

## 1 INTRODUCTION

Hepatocellular carcinoma (HCC) is identified as one of the main reasons of caner‐associated death in the world and half of death cases occur in China.^1^, ^2^ People have a high risk of developing HCC.^3^, ^4^ Although the oncological and surgical treatments have been improved in recent years, the prognosis of HCC patients is still not optimistic and the postoperative recurrence rate is high, resulting in that the survival rate of HCC patients is low.^1^, ^2^, ^5^, ^6^ It is well‐known that the molecular mechanisms underlying the biological processes of HCC are not well‐researched, which requires us to find a new lncRNA that may contribute to the diagnosis and prognosis of patients.

LncRNAs are a subgroup of non‐coding RNAs that have no or limited protein‐coding ability, consisting of more than 200 nucleotides.^7^ As lncRNAs exert their functions in many biological processes, their abnormal expressions are implicated in various cancers.^8^, ^9^ Many studies show that lncRNAs play important roles in the biological development of cancer cells, such as cell proliferation, migration, and EMT formation.^10^, ^11^, ^12^ More importantly, aberrant expression of lnc RNA TMPO antisense RNA 1 (TMPO‐AS1) promotes lung adenocarcinoma, prostate cancer.^8^, ^13^ The miR‐329‐3p is a critical miRNA and serves a tumor inhibitor in multiple cancers, such as suppressing cervical cancer cell proliferation.^14^, ^15^, ^16^, ^17^ Nevertheless, the role of TMPO‐AS1 and miR‐329‐3p in HCC has not been investigated before. Our interest is to find whether TMPO‐AS1/miR‐329‐3p axis modulates HCC development.

Therefore, in this study, we will be exploring the biological function of TMPO‐AS1in HCC progression, which might inspire us to find an effective treatment target for HCC.

## 2 MATERIALS AND METHODS

### 2.1 Tissue samples

About 48 HCC tissues and normal tissues were obtained from patients with HCC undergoing surgical resection at our hospital. Patients treated with radiotherapy or chemotherapy before study were excluded. Each participant signed the written informed consent. All fresh tissues were frozen in liquid nitrogen and stored immediately at −80°C. Ethical approval was obtained from the Ethics Committee of our hospital.

### 2.2 Cell culture and treatment

Human liver epithelial cell (THLE‐3) and HCC cells (Huh7, Hep3B, and LM3) were purchased from the Chinese Academy of Sciences (Beijing, China). Cells were incubated in RPMI‐1640 medium (Invitrogen, Carlsbad, CA, USA) containing 10% fetal bovine serum (FBS; Invitrogen) and 1% penicillin/streptomycin (Invitrogen) and cultured in a 5% CO_2_ incubator at 37°C. About 200 ng/mL of insulin‐like growth factor 1 (IGF‐1), the AKT/mTOR signaling pathway activator, was acquired from PeproTech (London, UK) to treat Huh7 and Hep3B cells.

### 2.3 Cell transfection

Huh7 and Hep3B cells were separately transfected with specific shRNAs against TMPO‐AS1 (sh‐TMPO‐AS1 #1#2) and the negative control (NC), as well as against pcDNA3.1/TMPO‐AS1, pcDNA3.1/FOXK1, and the empty pcDNA3.1 vector (all from GenePharma, Shanghai, China). The miR‐329‐3p mimics and NC mimics were obtained from GenePharma. Besides, the mock group also served as a control. Each mentioned plasmid was transfected into cells via Lipofectamine 2000 (Invitrogen).

### 2.4 Quantitative real‐time polymerase chain reaction (qRT‐PCR)

Total RNA was isolated from cells utilizing TRIzol reagent (Invitrogen) and was reverse‐transcribed into cDNA by Reverse Transcription Kit (Invitrogen). The qRT‐PCR was performed in the Bio‐Rad CFX96 system using SYBR‐Green Real‐Time Kit (Takara, Tokyo, Japan). The relative expression was normalized to GAPDH or U6 and fold expression changes were calculated with 2^‐ΔΔCt^ method.

### 2.5 Colony formation assay

1 × 10^3^ Huh7 and Hep3B cells were plated onto a fresh six‐well plate for 2‐week incubation. Formaldehyde (Sigma‐Aldrich) and crystal violet (Sigma‐Aldrich) were used for fixing and dying the colonies. The visible colonies were counted manually.

### 2.6 Cell counting kit‐8 (CCK‐8) assay

About 1 × 10^3^Hep3B and Huh7 cells were loaded in 96‐well plates and 10 μL of CCK‐8 solution (Dojindo, Kumamoto, Japan) was added and incubated for another 4 h. The absorbance at 450 nm was examined using a microplate Reader (Bio‐Rad).

### 2.7 Terminal‐deoxynucleoitidyl transferase‐mediated nick end labeling (TUNEL) assay

The apoptosis of Hep3B and Huh7cells were evaluated via TUNEL Apoptosis Kit (Invitrogen). DAPI (Koritai Biotechnology) was bought for nucleus staining. The cells were surveyed and captured through fluorescence microscopy (Olympus).

### 2.8 Transwell assay

Cell invasion and migration were evaluated using transwell chambers (Millipore) with or without Matrigel (Corning Inc, USA).Hep3B and Huh7cells were cultivated for 24 hours and then nonmigrated cells were removed. About 4% paraformaldehyde and crystal violet were used for fixing and staining the migrated cells and photographed by an IX71 inverted microscope (Olympus, Tokyo, Japan).About invasion assays, cells were incubated in serum‐free medium and seeded into the top compartment of the Matrigel‐coated plates, and the medium containing 10% FBS was added to the bottom compartment. After 48 hours, cells were fixed in 4% paraformaldehyde and then dyed using crystal violet. Finally, invaded or migrated cells were counted under a microscope (Olympus).

### 2.9 Subcellular fractionation assay

In order to determine cellular localization of TMPO‐AS1, cytoplasmic and nuclear fractions were collected via a Nuclear/ Cytoplasmic Isolation Kit (Norgenbiotek Corporation, Thorold, ON, Canada). Relative expression level of TMPO‐AS1 was determined by qRT‐PCR. GAPDH or U6 was the cytoplasmic control or nuclear control.

### 2.10 Western blot

Total protein was extracted with RIPA lysis buffer, isolated on SDS‐PAGE gel, and transferred to a PVDF membrane. Membranes were sealed with 5% skim milk and incubated overnight with primary antibodies for FOXK1(ab18196),E‐cadherin (ab15148), N‐cadherin (ab207608), MMP2 (ab97779), MMP7 (ab205525), p‐Akt (ab38449), Akt (ab179463), p‐mTOR (ab109268), mTOR (ab109268), and GAPDH (ab8245) from Abcam (Cambridge, USA). Secondary antibodies were added for cultivation for 1 hour. The amount of protein was examined using chemiluminescence detection system.

### 2.11 Animal studies

A total of 15 male BALB/c athymic nude mice (5‐week‐old, 17‐20 g), were brought from the National Laboratory Animal Center (Beijing, China) and were randomly divided into three groups. HCC xenografts in the nude mice were established by subcutaneously injecting with sh‐NC or sh‐TMPO‐AS1#1 transfected HCC cells. The untreated mice served as the mock group. Tumor volume was measured using a vernier caliper every 4 days utilizing the formula “volume = 1/2 × length ×width.” The mice were sacrificed after 28 days of injection, and the tumor was also weighed. This experiment was approved by the Ethics Committee of our hospital.

### 2.12 Luciferase reporter assay

The wild‐type (Wt) and mutant (Mut) binding sites of miR‐329‐3p in TMPO‐AS1 sequence or FOXK1 3′UTR was subcloned into pmirGLO dual‐luciferase vector to construct TMPO‐AS1‐Wt/Mut or FOXK1‐Wt/Wut and then mixed with specific transfection plasmids into Huh7 and Hep3B cells. The luciferase activity was determined using Dual‐Luciferase Reporter Assay System (Promega, MA, USA).

### 2.13 RNA pull down assay

The miR‐329‐3p‐Wt, miR‐329‐3p‐Mutand FOXK1‐Wt, FOXK1‐Mut was separately biotin labeled into Bio‐miR‐329‐3p‐Wt/Mut and Bio‐FOXK1‐Wt/Mut, with Bio‐NC as control. Then, cell lysates were incubated with the biotinylated probe and M‐280 streptavidin magnetic beads (Sigma‐Aldrich). The relative enrichment of miR‐641 or TMPO‐AS1 was analyzed by qRT‐PCR afterwards.

### 2.14 Statistical analysis

All statistical analyses were performed by SPSS (SPSS Inc, Chicago, IL, USA). Data were expressed as mean ± SD from more than three independent experiments. Student's t‐test and one‐way ANOVA were used to calculate the difference between two or more groups. The overall survival analysis was performed using Kaplan‐Meier method and gene correlation analysis was conducted by Pearson's correlation analysis method. P value of 0.05 or less was regarded as significance.

## 3 RESULTS

### 3.1 TMPO‐AS1‐regulated HCC cell proliferation, apoptosis, and invasion

**FIGURE 1:**
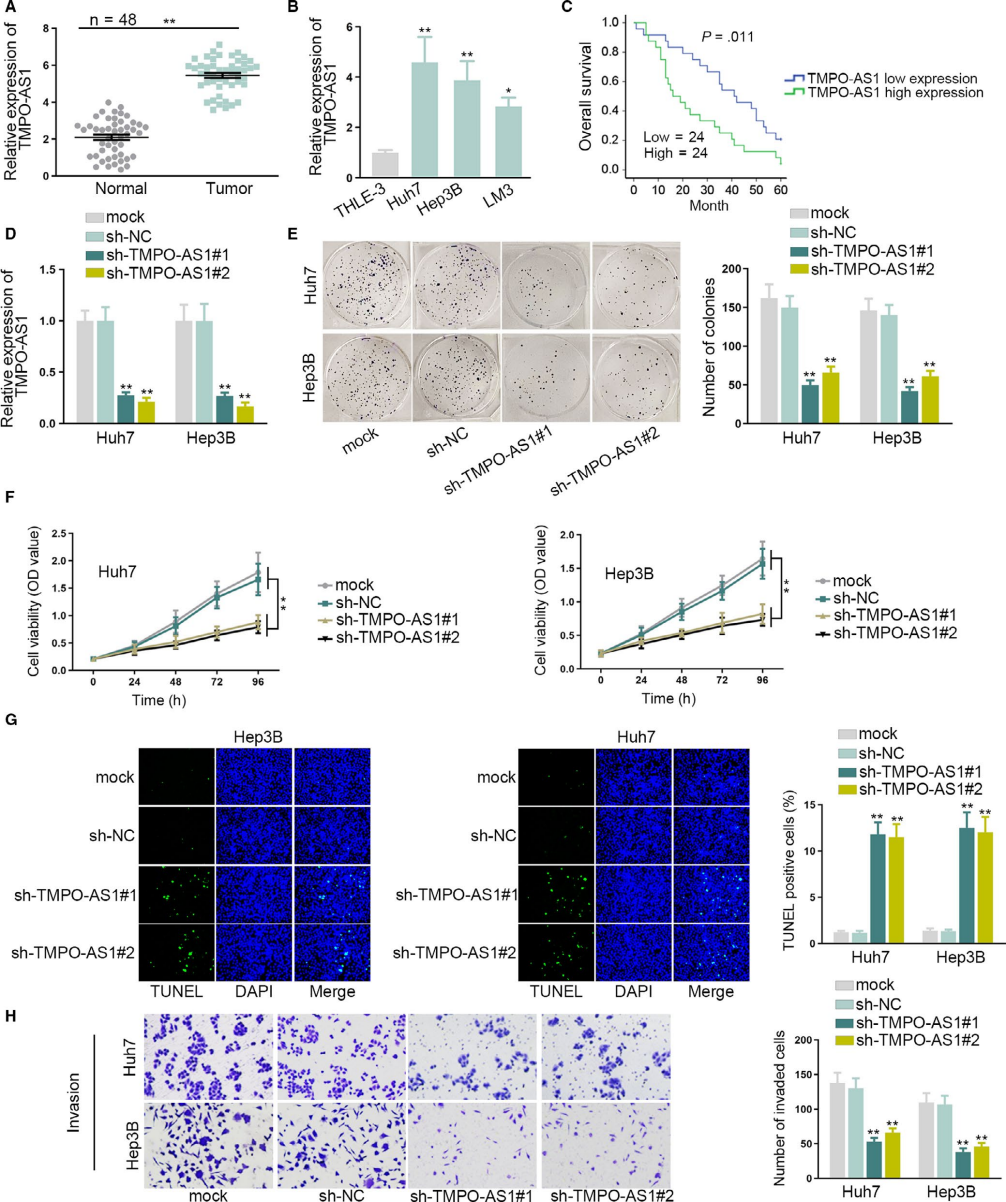
TMPO‐AS1 promoted proliferation and invasion of HCC cells. A, TMPO‐AS1expression in HCC tissues and normal liver tissues was measured by qRT‐PCR. B, The expression level of TMPO‐AS1in HCC cells and human liver epithelial cells was measured by qRT‐PCR. C, Kaplan‐Meier method was used to analyze the effect of TMPO‐AS1 expression on the survival time of HCC patients. D, TMPO‐AS1expression in sh‐TMPO‐AS1#1/2 transfected Huh7 and Hep3B cells was measured by qRT‐PCR. E, F, Colony formation and CCK‐8 assays tested Huh7 and Hep3B cell proliferation when silencing TMPO‐AS1. G, TUNEL assay assessed Huh7 and Hep3B cell apoptosis when downregulating TMPO‐AS1. H, Transwell assay detected Huh7 and Hep3B cell invasion when knocking down TMPO‐AS1.^*^ *P* < .05, ^**^ *P* < .01

To explore the role of TMPO‐AS1 in HCC, firstly, qRT‐PCR measured the expression of TMPO‐AS1 in HCC tissues and cells. TMPO‐AS1was upregulated in HCC tissues and cells (Huh7, Hep3B, and LM3) compared with normal liver tissues and human liver epithelial cell (THLE‐3) (Figure 1A,B). Additionally, Kaplan‐Meier analysis illustrated that the highTMPO‐AS1 expression was closely associated with poor prognosis of HCC patients (Figure 1C). Then the associations between TMPO‐AS1 and clinicopathological characteristics in patients with HCC were investigated. High TMPO‐AS1 expression was related to large tumor size, lymphatic metastasis, and advanced stage of cancer patients (Table 1).The knockdown efficiency of TMPO‐AS1 was measured in Huh7 orHep3B cells which contained higher expression of TMPO‐AS1than LM3 cells. TMPO‐AS1 expression was decreased by transfecting sh‐TMPO‐AS1#1/2 plasmids (Figure 1D). Next, several loss‐of‐function assays were performed. Results from colony formation and CCK‐8assays indicated that colony formation ability and cell viability in sh‐TMPO‐AS1#1/2 transfected Huh7 and Hep3B cells were decreased (Figure 1E,F). TUNEL assay detected that TUNEL‐positive cells were increased by knocking down TMPO‐AS1, indicating that cell apoptosis was stimulated. Furthermore, results from transwell assay showed that TMPO‐AS1downregulation decreased number of invaded and migrated cells (Figure 1H, Figure S1A). The western blot assay examined the expression of migration‐related proteins. When downregulating TMPO‐AS1, the expression of E‐cadherin was increased, and that of N‐cadherin, MMP2 as well as MMP7 was decreased, indicating that EMT process of HCC cells was obstructed by knocking downTMPO‐AS1 (Figure S1B). Additionally, animal studies were also carried out. Compared with control groups, the tumors obtained from mice injected with sh‐TMPO‐AS1#1 transfected cells were obviously smaller (Figure S1C). Besides, tumor volume and weight were also smaller and lighter than control groups (Figure S1D,E). The above results demonstrated that TMPO‐AS1 is involved in the development of HCC both in vitro and in vivo.

**Table 1:** Correlation between TMPO‐AS1 Expression and Clinical Features of hepatocellular carcinoma. (n = 48)

Variable	TMPO‐AS1 Expression		*P*‐value
	Low	High	
Age			
<55	5	8	.517
≥55	19	16	
Gender			
Male	10	12	.772
Female	14	12	
Tumor size			
<3	15	5	.008^**^
≥3	9	19	
Lymphatic metastasis			
No	13	4	.015^*^
Yes	11	20	
TNM			
I‐II	12	1	.0007^***^
III‐IV	12	23	

Note

Low/high by the sample median. Pearson χ^2^ test. ^*^ *P* < .05, ^**^ *P* < .01, ^***^ *P* < .001 was considered to be statistically significant.

### 3.2 TMPO‐AS1 sponged miR‐329‐3p in HCC cells

**FIGURE 2:**
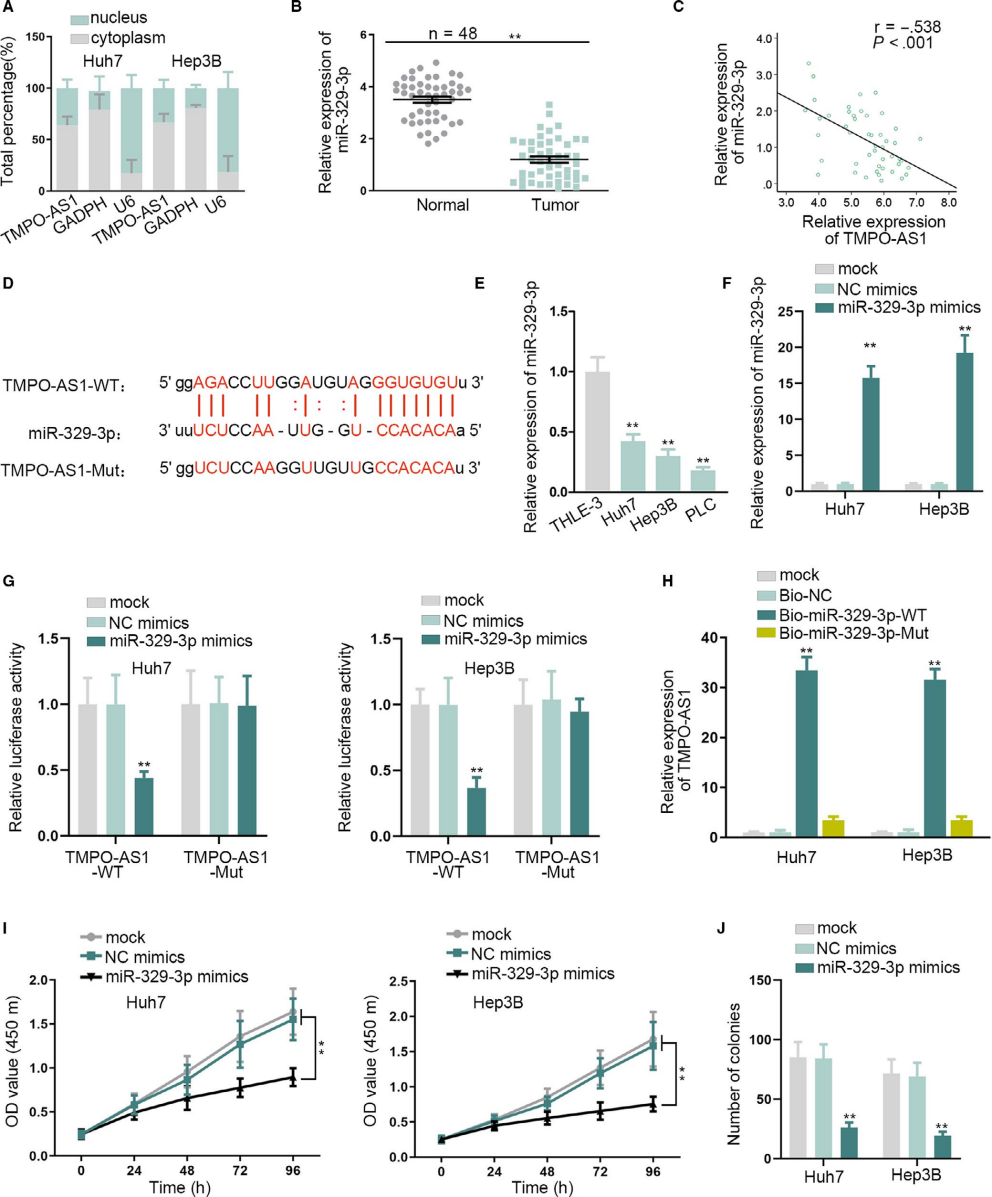
MiR‐329‐3p acted as a target of TMPO‐AS1 and it negatively correlated with TMPO‐AS1 expression. A, The subcellular fraction assay was to test the RNA localization. B, MiR‐329‐3p expression was assessed in HCC tissues and normal liver tissues by qRT‐PCR. C, Pearson's correlation analysis was performed to analyze the correlation between miR‐329‐3p and TMPO‐AS1in HCC tissues. D, The binding site between miR‐329‐3p and TMPO‐AS1 was predicted by starBase. E, qRT‐PCR measured the expression of miR‐329‐3p in HCC cells and THLE‐3 cells. F, qRT‐PCR detected the transfection efficiency of miR‐329‐3p mimics. G, H, Luciferase reporter and RNA pull down assays confirmed the binding relation between miR‐329‐3p and TMPO‐AS1. I, J, CCK‐8 and colony formation assays tested cell proliferation under the effect of miR‐329‐3p mimics. ^**^ *P* < .01

According to the result that TMPO‐AS1 mainly distributed in cytoplasm, confirmed by subcellular fractionation assay (Figure 2A).We speculated that TMPO‐AS1 possibly affects cancer progression via ceRNA regulation pattern. Subsequently, starBase was utilized to screen the miRNAs that might bind with TMPO‐AS1. After qRT‐PCR analyses, 29 miRNAs were downregulated in HCC cells and 51 miRNAs were negatively regulated by TMPO‐AS1.Finally two prequalified miRNAs (miR‐329‐3p and miR‐4500) were obtained by taking the intersection of two groups of miRNAs **(**Figure S2A). Then, the expression of miR‐329‐3p and miR‐4500 was tested in HCC tissues and normal tissues. The results of qRT‐PCR assay showed that the expression of miR‐4500 presented no obvious differences in HCC tissues and normal tissues, while miR‐329‐3p was significantly downregulated in HCC tissues **(**Figure S2BB, Figure 2B). Besides, Pearson's correlation analysis validated that miR‐329‐3p was negatively correlated with TMPO‐AS1 in HCC **(**Figure 2C). Through starBase, the binding site between miR‐329‐3p and TMPO‐AS1 was obtained (Figure 2D). Therefore, miR‐329‐3p was chosen as the object of the studies below. MiR‐329‐3p was less expressed in HCC cells compared with THLE‐3 cells (Figure 2E). Similarly, to explore the biological function of miR‐329‐3p in HCC,miR‐329‐3p overexpression efficiency was evaluated by transfecting miR‐329‐3p mimics into Huh7 and Hep3B cells, and the expression of miR‐329‐3p was increased (Figure 2F). Luciferase reporter assay detected that the luciferase activity of TMPO‐AS1‐WT was decreased by miR‐329‐3p mimics, and that of TMPO‐AS1‐Mut was not affected (Figure 2G). RNA pull down assay showed that expression of TMPO‐AS1was enriched in the Bio‐miR‐329‐3p‐WT group rather than the mock, Bio‐NC, or Bio‐miR‐329‐3p‐Mut group (Figure 2H). Additionally, CCK‐8 and colony formation assays demonstrated that overexpressing miR‐329‐3p inhibited cell proliferation (Figure 2I,J). In conclusion, TMPO‐AS1 could bind with miR‐329‐3p and miR‐329‐3p overexpression suppressed HCC cell proliferation.

### 3.3 Oncogenic function of FOXK1 in HCC cells is dependent on miR‐329‐3p

**FIGURE 3:**
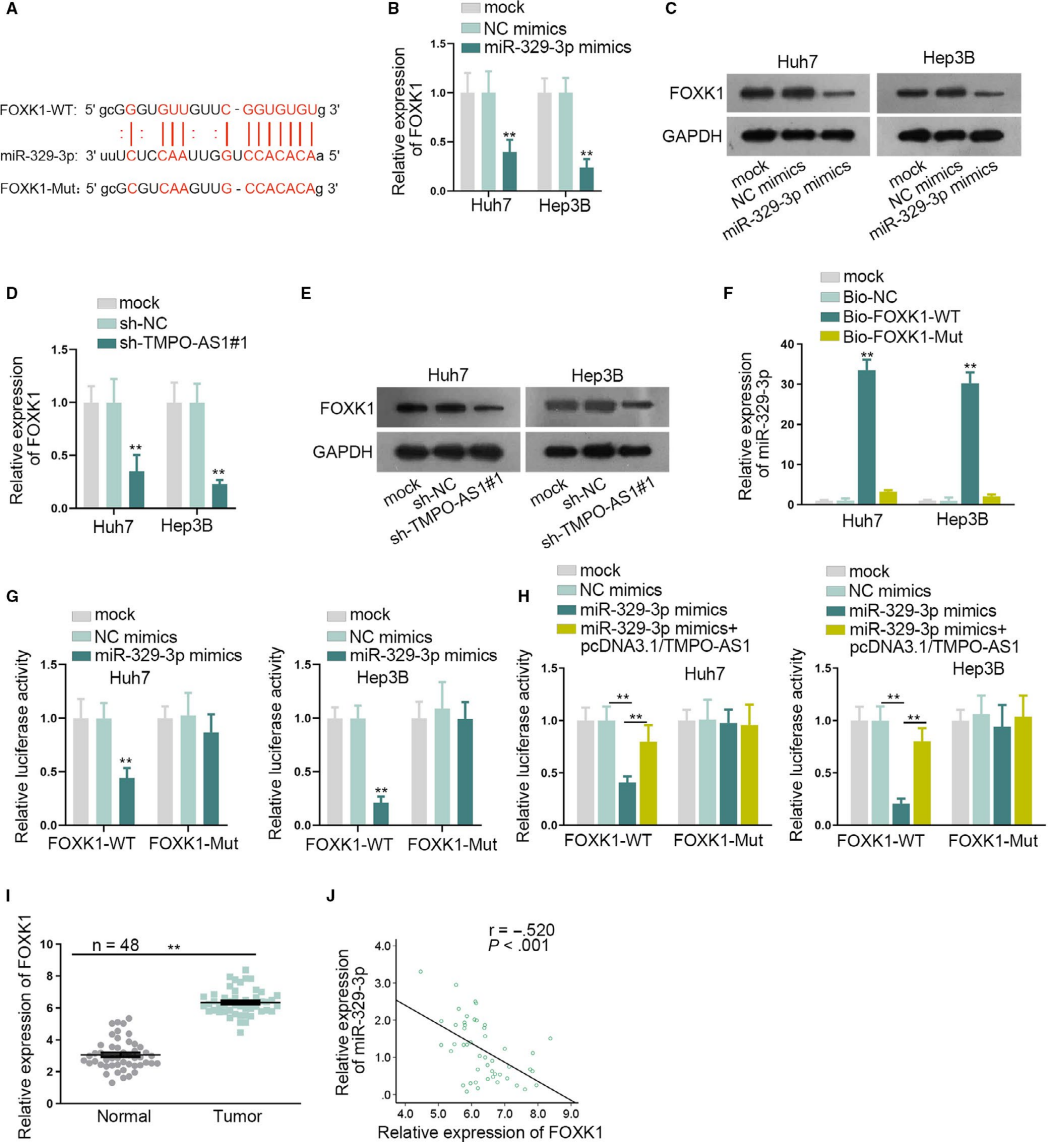
FOXK1 served as a target of miR‐329‐3p. A, The binding site between miR‐329‐3p and FOXK1 was predicted by starBase. B‐E, qRT‐PCR and western blot assays tested FOXK1mRNA and protein expressions affected by miR‐329‐3p mimics or sh‐TMPO‐AS1. F, G, RNA pull down and luciferase reporter assays verified the binding potential between FOXK1 and miR‐329‐3p. H, Luciferase reporter assay detected luciferase activity of FOXK1 in differently transfected groups. I, Expression of miR‐329‐3p in HCC tissues and normal liver tissues was evaluated by qRT‐PCR. J, Pearson's correlation analysis was used to prove the correlation between miR‐329‐3p and FOXK1. ^**^ *P* < .01

To find out the target gene of miR‐329‐3p, firstly, five possible downstream mRNAs of miR‐329‐3p were screened out through starBase (Figure S2C). qRT‐PCR assay confirmed that FOXK1was abnormally upregulated in HCC cells, yet the expression of other four mRNAs, such as ICE1 andAIF1L, did not exhibit significant differences in HCC cells compared with the THLE‐3 cells (Figure S2D). So, we focused on FOXK1 in the following assays. In a similar way, the binding site between FOXK1 and miR‐329‐3p was predicted by starBase (Figure 3A). qRT‐PCR and western blot assays measured that FOXK1mRNA and protein expressions declined when overexpressing miR‐329‐3p (Figure 3B,C). However, TMPO‐AS1 knockdown led to a decrease in the mRNA and proteinexpressions of FOXK1 (Figure 3D,E). RNA pull down assay examined that Bio‐FOXK1‐WT group was enriched with miR‐329‐3p, indirectly proving the binding relation between FOXK1 and miR‐329‐3p (Figure 3F). Afterward, luciferase reporter assay showed that the miR‐329‐3p mimics suppressed the luciferase activity of FOXK1‐WT rather that of FOXK1‐Mut (Figure 3G). Furthermore, TMPO‐AS1overexpression neutralized the suppressing effects of miR‐329‐3p mimics on luciferase activity of FOXK1‐WT (Figure 3H). Besides, FOXK1 was upregulated in HCC tissues compared with normal tissues (Figure 3I). As expected, FOXK1was negatively correlated with miR‐329‐3p in HCC tissues (Figure 3J). To sum up, FOXK1was the target gene of miR‐329‐3p, and TMPO‐AS1 could upregulate FOXK1 via competitive binding with miR‐329‐3p in HCC cells.

### 3.4 TMPO‐AS1 promotes HCC by regulating miR‐329‐3p/FOXK1 axis

**FIGURE 4:**
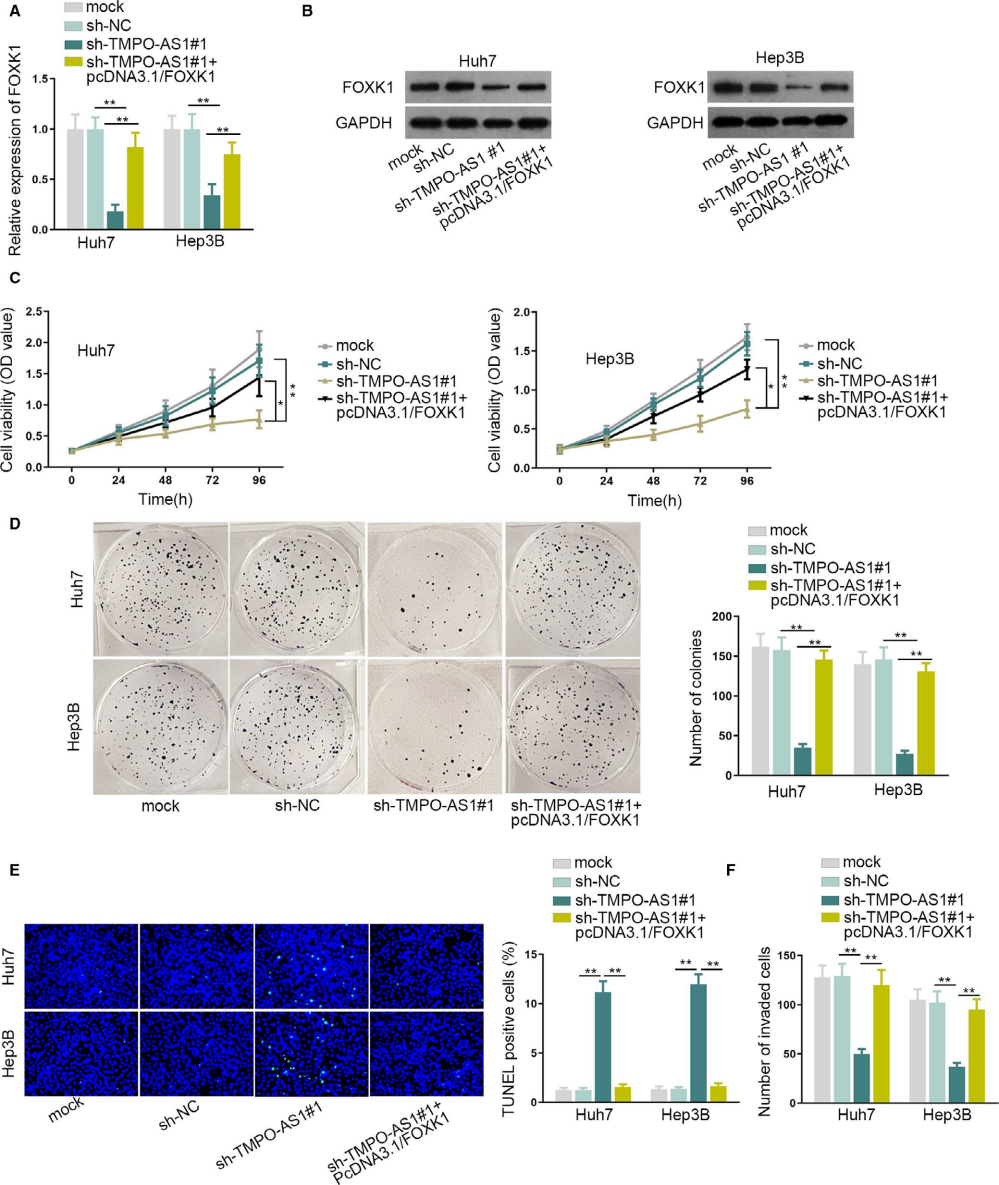
TMPO‐AS1 upregulated FOXK1 to promote HCC. A, B, qRT‐PCR and western blot assays were conducted to detect FOXK1mRNA and protein expressions in differently transfected groups. C, D, The capacity of cell proliferation was detected by CCK‐8 and colony formation assays. E, Cell apoptosis was detected by TUNEL assay. F, Cell invasion was tested by transwell assay.^*^ *P* < .05,^**^ *P* < .01

Previous assays showed that TMPO‐AS1, miR‐329‐3p, and FOXK1constituted a ceRNA network in HCC cells, so we proposed whetherTMPO‐AS1 affected the development of HCC via regulating miR‐329‐3p and FOXK1. Therefore, on one hand,miR‐329‐3p inhibitor was used in the rescue experiments. The knockdown efficiency of miR‐329‐3p was detected in miR‐329‐3p inhibitor transfected cells, and miR‐329‐3p expression was remarkably decreased (Figure S3A). Afterward, the results of the CCK‐8 and colony formation assays proved that miR‐329‐3p downregulation reversed the repressing effects of TMPO‐AS1 silence on cell proliferation (Figure S3B,C). The facilitating role of TMPO‐AS1 depletion on cell apoptosis was also neutralized by the miR‐329‐3p inhibitor (Figure S3D). In addition, when suppressing miR‐329‐3p expression, the impeditive effects of TMPO‐AS1 insufficiency on cell invasion, migration, and EMT were also counteracted (Figure S3E,F,G). On the other hand, we measured the overexpression efficiency of FOXK1 in HCC cells. qRT‐PCR and western blot assays showed that FOXK1 overexpression neutralized the inhibitory effect of TMPO‐AS1 depletion on mRNA and protein expressions of FOXK1 (Figure 4A,B). Proliferation assays detected that FOXK1 upregulation reversed the restraining influence of TMPO‐AS1 knockdown on cell proliferation (Figure 4C,D). TUNEL assay showed that FOXK1 upregulation offsets the accelerating effects of TMPO‐AS1 knockdown on cell apoptosis (Figure 4E). Furthermore, results from transwell and western blot assays elucidated that FOXK1 offsets the obstructive effects of TMPO‐AS1 deficiency on invasion, migration, and EMT process of HCC cells(Figure 4F, Figure S4E‐G).Overall, TMPO‐AS1 promoted HCC by regulating the miR‐329‐3p/FOXK1 axis.

### 3.5 TMPO‐AS1 promoted AKT/mTOR pathway in HCC cells by modulating FOXK1

**FIGURE 5:**
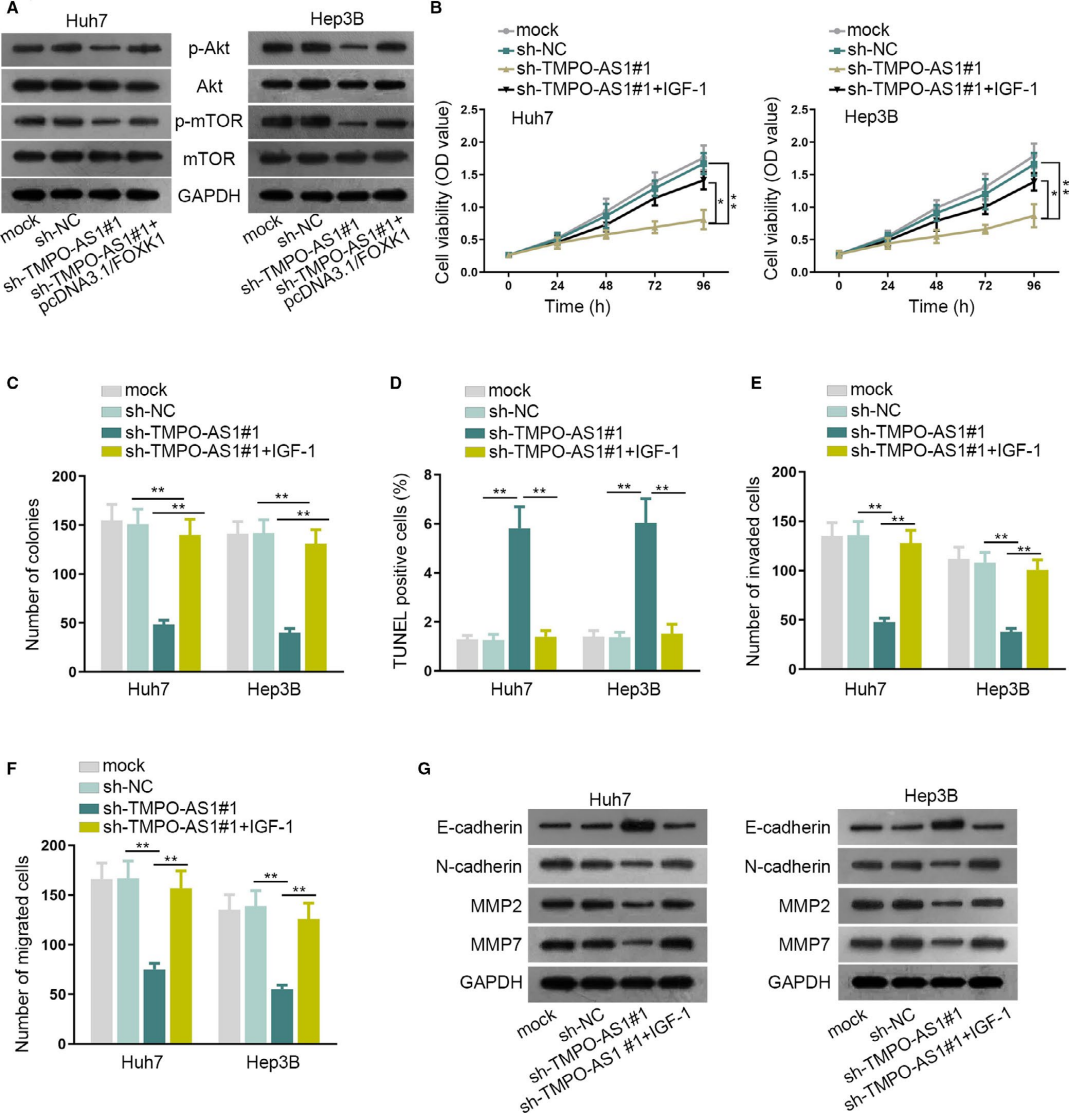
AKT/mTOR pathway was promoted by TMPO‐AS1 in HCC cells. A, Western blot assay assessed the level of AKT/mTOR pathway‐associated proteins in Huh7 and Hep3B cells. B, C, CCK‐8 and colony formation assays detected cell proliferation in different treatment groups. D‐F, TUNEL and transwell assays detected cell apoptosis and invasion/migration abilities respectively in different treatment groups. G, Western blot assay detected the level of EMT‐ and migration‐related proteins.^*^ *P* < .05, ^**^ *P* < .01

Several papers have reported that FOXK1 could regulate the AKT/mTOR pathway.^18^, ^19^, ^20^ Therefore, we tried to investigate whetherTMPO‐AS1 could modulate AKT/mTOR pathway via FOXK1in HCC cells. Firstly, western blot assay examined the expression of AKT/mTOR pathway‐related proteins. TMPO‐AS1 knockdown markedly decreased the p‐AKT and p‐mTOR expressions in Huh7 and Hep3Bcells. Moreover, this inhibitory effect of sh‐TMPO‐AS1was then offset by FOXK1 upregulation, indicating that TMPO‐AS1 exerted the underlying regulatory function on AKT/mTOR pathway (Figure 5A). Subsequently, rescue assays were performed to verify our assumptions with using IGF‐1, the AKT/mTOR pathway activator. Results of CCK‐8, colony formation and TUNEL assay suggested that IGF‐1 treatment reversed the suppressing effects of TMPO‐AS1depletion on cell proliferation as well as the encouraging effects of that on cell apoptosis (Figure 5B‐D). Transwell and western blot assays showed that the interruptive effects of TMPO‐AS1depletion on cell invasion, migration and EMT were remedied by IGF‐1 treatment (Figure 5E‐G). These results showed that TMPO‐AS1 facilitates HCC development through upregulating FOXK1 expression to stimulate AKT/mTOR pathway.

## 4 DISCUSSION

Many papers have documented that lncRNAs play crucial roles in HCC progression. For illustration, lncRNA n339260 facilitates vasculogenic mimicry and strengthens cancer stem cell in HCC.^21^ The lncRNA SchLAH represses HCC metastasis via interacting with FUSin sarcoma.^22^ Also, lncRNAs can serve as therapeutic targets for HCC patients. For instance, lncRNA AWPPH contributes to the development of HCC progression by YBX1 and acts as a prognostic marker.^23^ The lncRNA CCHE1 suggests a bad prognosis of HCC and promotes HCC progression by activating the ERK/MAPK pathway.^24^ In our research, TMPO‐AS1 was upregulated in HCC tissues and cell lines. Besides, the oncogenic role of TMPO‐AS1 has been confirmed in cancers, such as cervical cancer^25^ and nonsmall cell lung cancer.^11^ Furthermore, TMPO‐AS1 is also identified as an underlying therapeutic target for patients diagnosed with cancers, including colorectal cancer,^26^ prostate cancer,^10^ and lung adenocarcinoma.^27^ Therefore, we conjectured whether TMPO‐AS1 also contributed to the development of HCC, and inhibitingTMPO‐AS1 benefited the improvement of the prognosis of HCC patients. Our research found that high TMPO‐AS1 expression was closely associated with unsatisfactory prognosis of HCC patients. The results of the functional assays demonstrated that TMPO‐AS1 depletion suppressed cell proliferation, invasion, migration, and EMT process, meanwhile, inhibited tumor growth, suggesting the tumor promoter role of TMPO‐AS1 in HCC.

Moreover, to figure out whether TMPO‐AS1 regulated HCC via ceRNA pattern, the downstream miRNAs of TMPO‐AS1 were explored. In the present study, miR‐329‐3p was screened by bioinformatics tool and mechanism experiments. Besides, miR‐329‐3p was certified as a tumor suppressor in cervical cancer,^14^, ^15^ and the number of related researches about its role in cancers is limited. In this study, miR‐329‐3p overexpression offsets the repressing influence of TMPO‐AS1 depletion on HCC cell growth. Subsequently, the decisive mRNA in ceRNA network, FOXK1 was explored. According to previous researches, FOXK1 promotes liver cancer,^19^ breast cancer,^28^ esophageal cancer,^29^ colorectal cancer,^30^ prostate cancer^31^ etc. Whether TMPO‐AS1 could modulate HCC progression by targeting FOXK1/miR‐329‐3p axis was worth investigating. Additionally, the regulatory effects of FOXK1on AKT/mTOR pathway have been elucidated by other investigations.^18^, ^19^, ^20^ Hence, whetherTMPO‐AS1 could stimulate AKT/mTOR pathway via regulating FOXK1 to promote HCC further interested us. Through western blot assays, TMPO‐AS1 knockdown obviously inhibited p‐Akt and p‐mTOR expressions, and this inhibitory influence was counteracted by FOXK1 upregulation. In addition, IGF‐1 (an activator of AKT/mTOR pathway) treatment could reverse the obstructive effects of TMPO‐AS1 knockdown on cell proliferation, invasion, migration, and EMT process, indicating the significant influence of AKT/mTOR pathway stimulation on the regulatory role of TMPO‐AS1 in HCC.

In a summary, TMPO‐AS1 promotes HCC through sponging miR‐329‐3p to upregulate FOXK1 and activate AKT/mTOR signaling pathway, which was the first to illustrate the potential of TMPO‐AS1 as a therapeutic target in HCC. In addition, the upstream molecular mechanism of TMPO‐AS1 in HCC cells waits to be explored in our further studies.

## CONFLICT OF INTEREST

No conflict of interest was involved in this study.

## AUTHOR CONTRIBUTIONS

Xiaobo Guo contributed to experimental design and took part in drafting the manuscript and revision, Yun Wang performed the experiments. They both recorded the results in all experiments.

## Supporting information

Fig S1

Fig S2

Fig S3

Fig S4

## Data Availability

Not applicable.

## References

[1] Jiang J‐F , Lao Y‐C , Yuan B‐H , et al. Treatment of hepatocellular carcinoma with portal vein tumor thrombus: advances and challenges. Oncotarget. 2017;8(20):33911‐33921. 28430610 10.18632/oncotarget.15411 PMC5464922

[2] Hartke J , Johnson M , Ghabril M . The diagnosis and treatment of hepatocellular carcinoma. Semin Diagn Pathol. 2017;34(2):153‐159. 28108047 10.1053/j.semdp.2016.12.011

[3] Gravitz L . Liver cancer. Nature. 2014;516(7529):S1. 25470192 10.1038/516S1a

[4] Kelly D , Sharif K , Brown RM , Morland B . Hepatocellular carcinoma in children. Clin Liver Dis. 2015;19(2):433‐447. 25921672 10.1016/j.cld.2015.01.010

[5] Bruix J , Han KH , Gores G , Llovet JM , Mazzaferro V . Liver cancer: approaching a personalized care. J Hepatol. 2015;62(1 Suppl):S144‐S156. 25920083 10.1016/j.jhep.2015.02.007 PMC4520430

[6] Grandhi MS , Kim AK , Ronnekleiv‐Kelly SM , Kamel IR , Ghasebeh MA , Pawlik TM . Hepatocellular carcinoma: from diagnosis to treatment. Surg Oncol. 2016;25(2):74‐85. 27312032 10.1016/j.suronc.2016.03.002

[7] Ng SY , Lin L , Soh BS , Stanton LW . Long noncoding RNAs in development and disease of the central nervous system. Trends Genet. 2013;29(8):461‐468. 23562612 10.1016/j.tig.2013.03.002

[8] Li J , Li Z , Zheng W , et al. LncRNA‐ATB: An indispensable cancer‐related long noncoding RNA. Cell Prolif. 2017;50(6):e12381. 10.1111/cpr.12381 PMC6529097 28884871

[9] Botti G , Marra L , Malzone M , et al. LncRNA HOTAIR as prognostic circulating marker and potential therapeutic target in patients with tumor diseases. Curr Drug Targets. 2017;18(1):27‐34. 26648066 10.2174/1389450117666151209122950

[10] Huang W , Su X , Yan W , et al. Overexpression of AR‐regulated lncRNA TMPO‐AS1 correlates with tumor progression and poor prognosis in prostate cancer. Prostate. 2018;78(16):1248‐1261. 30105831 10.1002/pros.23700

[11] Qin Z , Zheng X , Fang Y . Long noncoding RNA TMPO‐AS1 promotes progression of non‐small cell lung cancer through regulating its natural antisense transcript TMPO. Biochem Biophys Res Comm. 2019;516(2):486‐493. 31230752 10.1016/j.bbrc.2019.06.088

[12] Li DS , Ainiwaer JL , Sheyhiding I , Zhang Z , Zhang LW . Identification of key long non‐coding RNAs as competing endogenous RNAs for miRNA‐mRNA in lung adenocarcinoma. Eur Rev Med Pharmacol Sci. 2016;20(11):2285‐2295. 27338053

[13] Martens‐Uzunova ES , Bottcher R , Croce CM , Jenster G , Visakorpi T , Calin GA . Long noncoding RNA in prostate, bladder, and kidney cancer. Eur Urol. 2014;65(6):1140‐1151. 24373479 10.1016/j.eururo.2013.12.003

[14] Chang YH , Yin F , Fan GF , Zhao M . Down‐regulation of miR‐329‐3p is associated with worse prognosis in patients with cervical cancer. Eur Rev Med Pharmacol Sci. 2017;21(18):4045‐4049. 29028098

[15] Li W , Liang J , Zhang Z , et al. MicroRNA‐329‐3p targets MAPK1 to suppress cell proliferation, migration and invasion in cervical cancer. Oncol Rep. 2017;37(5):2743‐2750. 28393232 10.3892/or.2017.5555

[16] Xu J , Zhang J . LncRNA TP73‐AS1 is a novel regulator in cervical cancer via miR‐329‐3p/ARF1 axis. J Cell Biochem. 2019 121(1):344–352. 31232491 10.1002/jcb.29181

[17] Daniel R , Wu Q , Williams V , Clark G , Guruli G , Zehner Z . A panel of microRNAs as diagnostic biomarkers for the identification of prostate cancer . Int J. Mol Sci. 2017;18(6):1281. 10.3390/ijms18061281 PMC5486103 28621736

[18] Yang H , Song Z , Wu X , Wu Y , Liu C . MicroRNA‐652 suppresses malignant phenotypes in glioblastoma multiforme via FOXK1‐mediated AKT/mTOR signaling pathway. Onco Targets Therapy. 2019;12:5563‐5575. 10.2147/OTT.S204715 PMC6630095 31371994

[19] Cui H , Gao Q , Zhang L , Han F , Wang L . Knockdown of FOXK1 suppresses liver cancer cell viability by inhibiting glycolysis. Life Sci. 2018;213:66‐73. 30312701 10.1016/j.lfs.2018.10.018

[20] Liu F , Liu S , Ai F , et al. miR‐107 promotes proliferation and inhibits apoptosis of colon cancer cells by targeting prostate apoptosis response‐4 (Par4). Oncol Res. 2017;25(6):967‐974. 27938501 10.3727/096504016X14803476672380 PMC7841080

[21] Zhao X , Sun B , Liu T , et al. Long noncoding RNA n339260 promotes vasculogenic mimicry and cancer stem cell development in hepatocellular carcinoma. Cancer Sci. 2018;109(10):3197‐3208. 30022558 10.1111/cas.13740 PMC6172069

[22] Ge Z , Cheng Z , Yang X , et al. Long noncoding RNA SchLAH suppresses metastasis of hepatocellular carcinoma through interacting with fused in sarcoma. Cancer Sci. 2017;108(4):653‐662. 28196303 10.1111/cas.13200 PMC5406589

[23] Zhao X , Liu Y , Yu S . Long noncoding RNA AWPPH promotes hepatocellular carcinoma progression through YBX1 and serves as a prognostic biomarker. Biochim Biophys Acta. 2017;1863(7):1805‐1816. 10.1016/j.bbadis.2017.04.014 28428004

[24] Peng W , Fan H . Long noncoding RNA CCHE1 indicates a poor prognosis of hepatocellular carcinoma and promotes carcinogenesis via activation of the ERK/MAPK pathway. Biomed Pharmacother. 2016;83:450‐455. 27427851 10.1016/j.biopha.2016.06.056

[25] Yang J , Liang B , Hou S . TMPO‐AS1 promotes cervical cancer progression by upregulating RAB14 via sponging miR‐577. J Gene Med. 2019;21(11):e3125. 31483914 10.1002/jgm.3125

[26] Mohammadrezakhani H , Baradaran B , Shanehbandi D , et al. Overexpression and clinicopathological correlation of long noncoding RNA TMPO‐AS1 in colorectal cancer patients. J Gastroint Cancer. 2019 10.1007/s12029-019-00333-7 31768869

[27] Peng F , Wang R , Zhang Y , et al. Differential expression analysis at the individual level reveals a lncRNA prognostic signature for lung adenocarcinoma. Mol Cancer. 2017;16(1):98. 28587642 10.1186/s12943-017-0666-z PMC5461634

[28] Gao F , Tian J . FOXK1, regulated by miR‐365‐3p, promotes cell growth and EMT indicates unfavorable prognosis in breast cancer. OncoTargets Therapy. 2020;13:623‐634. 32021304 10.2147/OTT.S212702 PMC6982530

[29] Chen DI , Wang K , Li X , et al. FOXK1 plays an oncogenic role in the development of esophageal cancer. Biochem Biophys Res Comm. 2017;494(1–2):88‐94. 29050933 10.1016/j.bbrc.2017.10.080

[30] Wang J , Liu G , Liu M , et al. The FOXK1‐CCDC43 Axis Promotes the Invasion and Metastasis of Colorectal Cancer Cells. Cell Physiol Biochem. 2018;51(6):2547‐2563. 30562730 10.1159/000495924

[31] Chen F , Xiong W , Dou K , Ran Q . Knockdown of FOXK1 Suppresses Proliferation, Migration, and Invasion in Prostate Cancer Cells. Oncol Res. 2017;25(8):1261‐1267. 28267429 10.3727/096504017X14871164924588 PMC7841013

