# Supplementary figures and images for: LncRNA TMPO‐AS1 promotes hepatocellular carcinoma cell proliferation, migration and invasion through sponging miR‐329‐3p to stimulate FOXK1‐mediated AKT/mTOR signaling pathway
Source: Cancer Med. 2020 May 27;9(14):5235–46. doi: 10.1002/cam4.3046 (PMC7367632; doi:10.1002/cam4.3046)

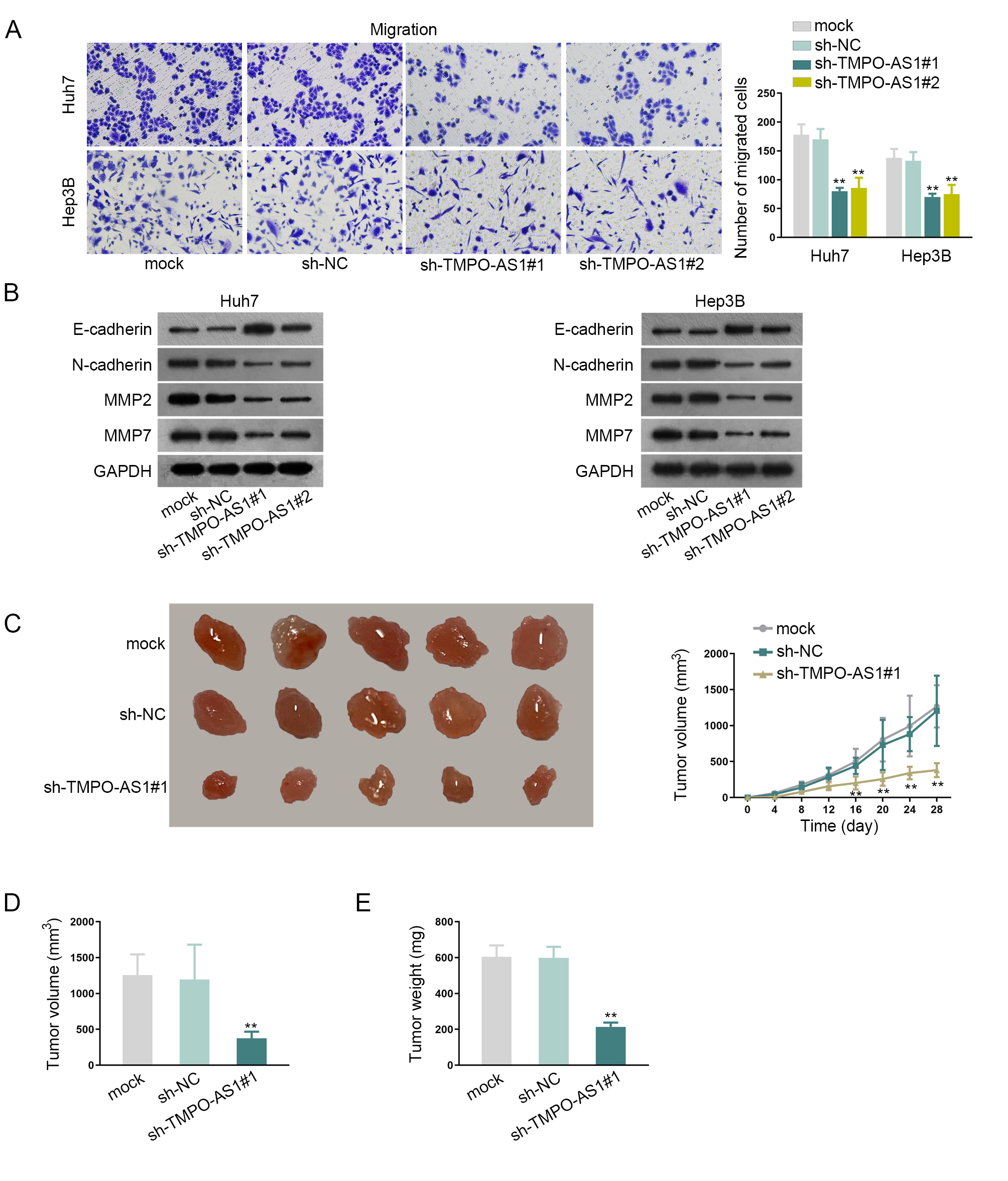

Supplement: Supplementary file 1 — Fig S1 [file CAM4-9-5235-s001.tif]

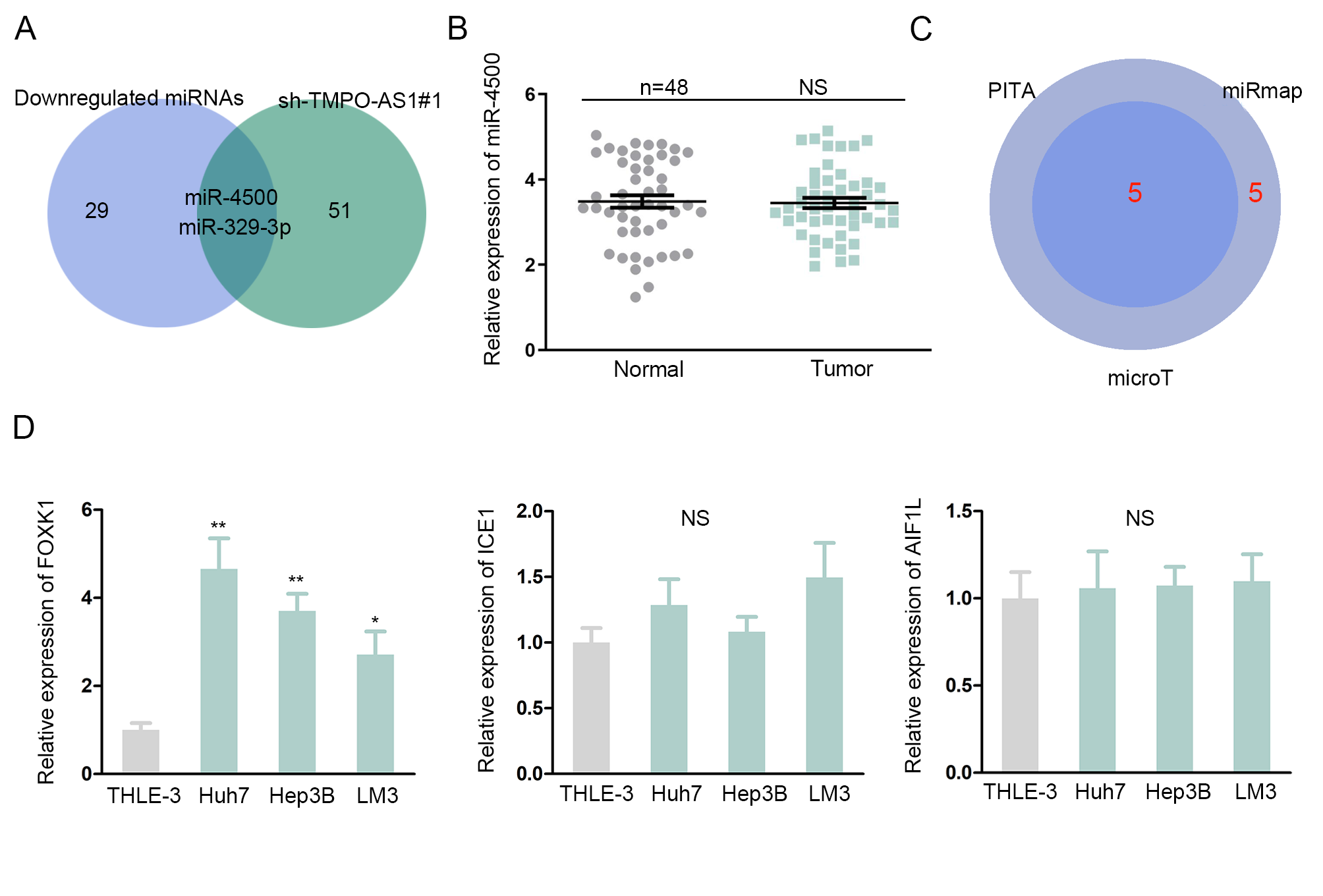

Supplement: Supplementary file 2 — Fig S2 [file CAM4-9-5235-s002.tif]

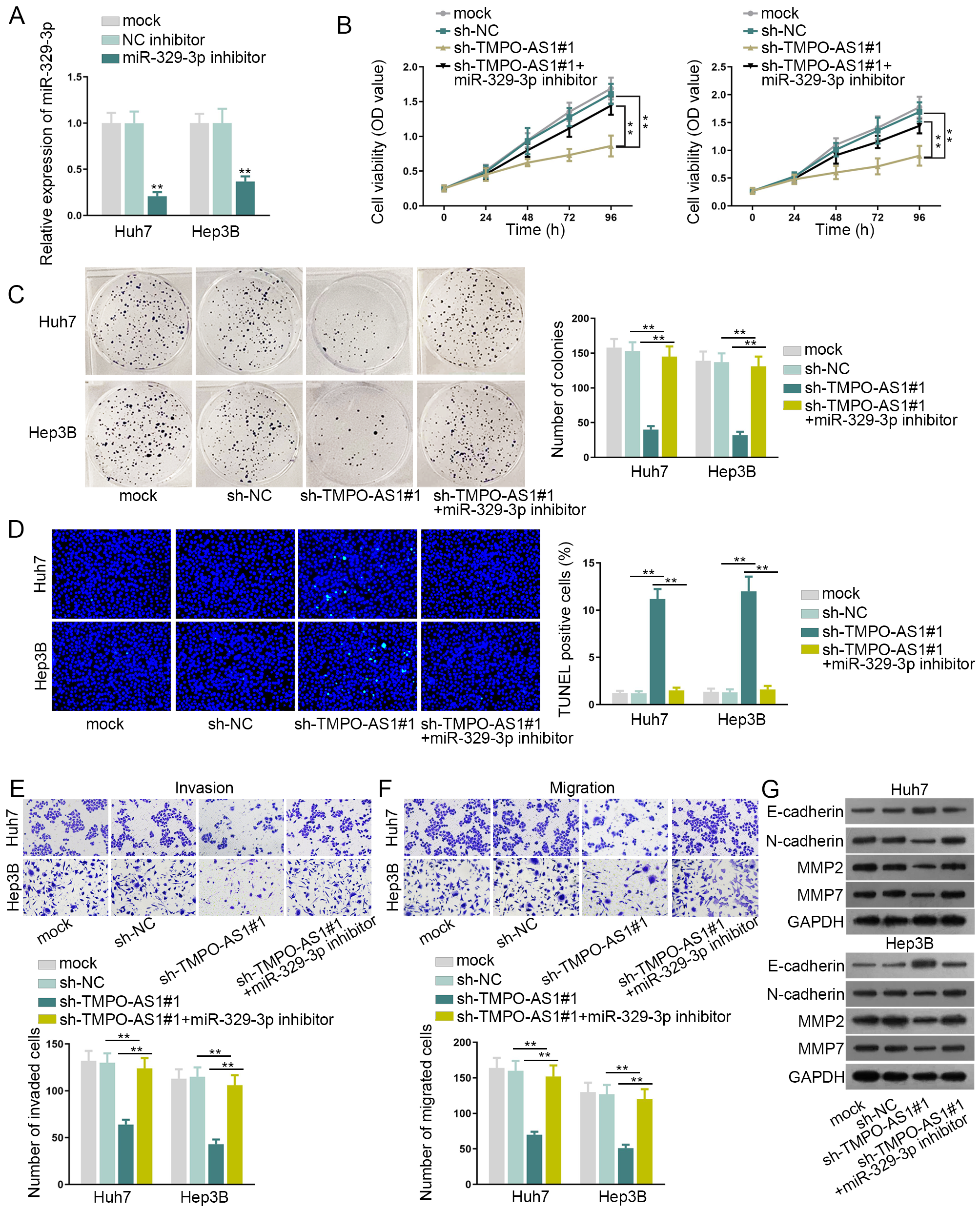

Supplement: Supplementary file 3 — Fig S3 [file CAM4-9-5235-s003.tif]

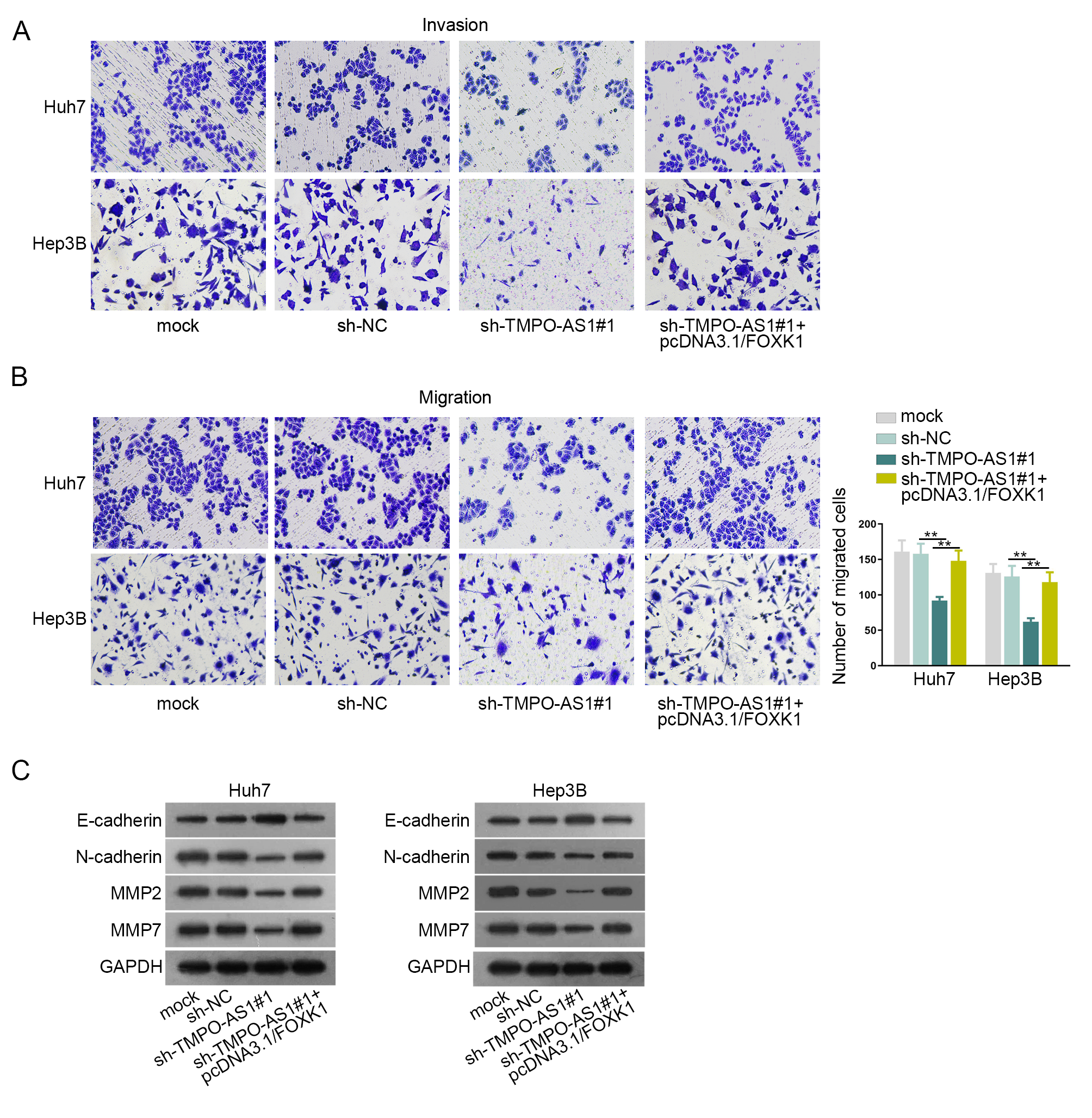

Supplement: Supplementary file 4 — Fig S4 [file CAM4-9-5235-s004.tif]
